# Risks of the ketogenic diet in CKD – the con part

**DOI:** 10.1093/ckj/sfad274

**Published:** 2023-11-07

**Authors:** Shivam Joshi, Rachel Shi, Jason Patel

**Affiliations:** Department of Veterans Affairs, Orlando, FL, USA; Department of Medicine, New York University Grossman School of Medicine, New York, NY, USA (Adjunct Faculty); University of Central Florida College of Medicine, Orlando, FL, USA; University of Arizona College of Medicine – Phoenix, Phoenix, AZ, USA

**Keywords:** ketogenic diet, kidney disease

## Abstract

The ketogenic diet is a very low carbohydrate diet that has received a lot of attention for its role in the treatment of type 2 diabetes and obesity. For patients with chronic kidney disease, there is limited evidence on the risks and/or benefits of this diet. However, from the limited evidence that does exist, there are several inferences that can be drawn regarding this diet for patients with kidney disease. The ketogenic diet may not be better than comparator higher carbohydrate diets over the long term. The diet also has low adherence levels in studies lasting ≥12 months. The diet's emphasis on fat, which often comes from animal fat, increases the consumption of saturated fat, which may increase the risk of heart disease. It has the potential to worsen metabolic acidosis by increasing dietary acid load and endogenous acid production through the oxidation of fatty acids. In addition, the diet has been associated with an increased risk of kidney stones in patients using it for the treatment of refractory epilepsy. For these reasons, and for the lack of safety data on it, it is reasonable for patients with kidney disease to avoid utilizing the ketogenic diet as a first-line option given alternative dietary patterns (like the plant-dominant diet) with less theoretical risk for harm. For those adopting the ketogenic diet in kidney disease, a plant-based version of the ketogenic diet may mitigate some of the concerns with animal-based versions of the ketogenic diet.

## INTRODUCTION

The ketogenic diet is a very low carbohydrate diet that was first used for the treatment of pediatric epilepsy in 1921 but has been recently popularized for the treatment of type 2 diabetes (T2D) and obesity. The diet prominently features fat as the main macronutrient (Table 1). Despite the diet's popularity in low carbohydrate circles, concerns have been raised regarding the safety, effectiveness and practicality of the diet over the long term [1, 2]. Patients with chronic kidney disease (CKD) may be more susceptible to some of the potential side effects of the diet, including metabolic acidosis and the unfavorable increase in low-density lipoprotein cholesterol (LDL-C) and apolipoprotein-B (apo-B). Nephrolithiasis is also a concern with this diet. Given the numerous alternative options with less potential risk and more evidence of safety, it is reasonable for patients with CKD to choose diets like the Mediterranean or the plant-dominant (PLADO) [3] diet before adopting a ketogenic diet.

**Table 1:** Macronutrient composition of the ketogenic diet.

Macronutrient	Total daily calories (%)
Fat	>70
Protein	6–20
Carbohydrate	<10

## KETOGENIC DIET IN T2D AND OBESITY

For highly adherent individuals, anecdotes of success abound in popular and social media. Support for the diet can also be found in the scientific literature. Indeed, several meta-analyses of short-term (<12 months) studies of the ketogenic diet, when compared with control diets, have shown significant clinical reductions in hemoglobin A1c and weight with the diet [4, 5]. However, several meta-analyses of long-term studies (≥12 months) have shown little to no statistically significant benefit with the ketogenic diet over comparator higher carbohydrate diets regarding weight loss or hemoglobin A1c [4–6].

A closer examination at the dwindling benefit observed in longer-term studies reveals that participants had poor adherence to the low-carbohydrate requirement of the ketogenic diet [4, 6]. For example, in one meta-analysis, nearly all the studies with data available reported participants on the ketogenic diet consuming a carbohydrate content *in excess* of the maximum amount of carbohydrates allowed for the ketogenic diet (typically defined as 10% of calories as carbohydrates or 50 g/day) by the end of the study period, thus precluding ketosis. The restrictive and unfamiliar nature of the diet may contribute to the difficulty with adherence.

Studies that have assessed adherence have shown better outcomes in those who adhere to the ketogenic diet [6], and adherence may be augmented with support (e.g. behavioral therapy, dietary counseling etc.). However, for weight loss, it has been previously demonstrated (repeatedly) that dietary adherence to caloric restriction, regardless of dietary type, is a more important determinant of success than the type of diet [7, 8]. By extension, weight loss in patients with insulin resistance will improve markers of glycemic control.

It is important to note several unique aspects of this diet in regard to weight loss and carbohydrate metabolism. First, some of the weight loss seen with the ketogenic diet may be due to nonfat loss, including muscle mass and body water losses [9]. Some studies have shown more fat loss with those eating higher-carbohydrate, plant-based diets when compared with the ketogenic diet [10]. Second, high dietary fat consumption as seen in the ketogenic diet may worsen carbohydrate metabolism when carbohydrates are reintroduced into the diet in significant quantities [2, 11].

## EVIDENCE IN KIDNEY DISEASE

The ketogenic diet has not been shown to negatively affect kidney function in at least four studies lasting 3–12 months [12–15]. However, the studies had limited follow-up, sizeable dropout rates or suboptimal adherence, all of which limit the ability to draw firm conclusions from these studies. Nonetheless, it appears that the ketogenic diet may not be harmful for kidney function in the short term, but further research is needed.

However, there is some evidence to suggest caution with the diet over the long term. Several studies have shown associations between albuminuria and the consumption of saturated fat and animal fat [16, 17], which are featured prominently in many popular iterations of the ketogenic diet. Another concern with the diet is the limited consumption of fiber-rich plant foods that are often excluded from the diet due to their higher carbohydrate content. Avoidance of these foods may preclude patients with CKD from obtaining some of benefits of these foods [18].

## DYSLIPIDEMIA

The effects of a ketogenic diet on cardiovascular risk factors of serum LDL-C, apo-B and triglycerides are variable and may be related to the fat and carbohydrate content and quality of the diet interventions, genetics, neurohormonal responses and patient adherence to the diet. While a ketogenic diet may decrease serum triglycerides in patients with T2D [19], more evidence from meta-analyses of randomized controlled trials suggests that ketogenic diets increase LDL-C and apo-B, which are more clearly associated with cardiovascular risk and atherosclerotic plaque growth [2, 20–22]. Although the effects on LDL-C are variable, with some studies reporting no difference in LDL-C [23], some individuals may experience extreme increases in their lipid profiles, as studies have reported variable increases in LDL-C, with a range of 5–10% in one trial to 44% (range 5–107%) in another trial [9]. Increases in LDL-C may be related to high intake of saturated and naturally occurring trans fat, low levels of polyunsaturated fatty acids (PUFAs) and monounsaturated fatty acids (MUFAs) and low levels of fiber in the ketogenic diet [9]. Both small and large LDL-C particles appear to be highly atherogenic [24] and therefore the improvements in triglycerides may be outweighed by the cardiovascular risks associated with increases in apo-B and LDL-C. These findings are of concern since patients with CKD are already at an increased risk for cardiovascular disease [25].

In addition, ketogenic diets emphasize the consumption of animal proteins and high fat dairy products that contain more cholesterol, saturated fat and naturally occurring trans fat. On the other hand, plant proteins and plant fats not only contain more PUFAs and MUFAs, which tend to reduce LDL-C levels [9], but also fiber and antioxidants, which may lower blood pressure and arterial inflammation and enhance gut microbiome diversity [26]. Relatively carbohydrate-dense plant foods such as legumes, fruits, oats and whole grains are associated with a reduced cardiovascular disease mortality and all-cause mortality [27] and contain more soluble fiber, which may reduce absorption of cholesterol in the gut and improve serum LDL-C, apo-B and triglycerides [28]. These potentially beneficial foods are often restricted in ketogenic diets.

## METABOLIC ACIDOSIS

The ketogenic diet can lead to metabolic acidosis through several mechanisms. The low or potentially low consumption of fruits and vegetables can lead to reduced dietary alkali intake [29]. Dietary alkali is often found in carbohydrate-rich plant foods in the form of citrate, malate and bicarbonate. Second, popular versions of the ketogenic diet often utilize animal-based proteins and fats, which, in contrast to plant foods, often have high amounts of dietary acids. Third, metabolic acidosis from ketone production and the oxidation of fatty acids can also contribute to reduced urinary citrate [30]. Patients with advanced CKD are less able to tolerate these changes due to a decrease in the kidney's capacity to excrete acid through reduced urinary ammonium excretion [31]. Together, these factors can lead to metabolic acidosis, which can result in significant downstream complications.

For patients with CKD, untreated metabolic acidosis has been shown to hasten the decline of renal function in a meta-analysis of studies using oral alkali for the treatment of metabolic acidosis [32]. In one prominent study by Goraya *et al.* [33], participants used fruits and vegetables for the treatment of metabolic acidosis and were able to slow the progression of CKD compared with those with untreated metabolic acidosis after 5 years. Patients consuming ketogenic diets may worsen their metabolic acidosis for the reasons mentioned previously and, as a result, may hasten the decline of their kidney function. In addition, uncontrolled metabolic acidosis may have other effects on the body, including the development or worsening of renal bone disease, muscle wasting, hypoalbuminemia, muscle inflammation, protein malnutrition and even mortality [31].

## NEPHROLITHIASIS

Another potential complication of metabolic acidosis in patients consuming ketogenic diets is nephrolithiasis. In studies of pediatric patients utilizing a ketogenic diet for the treatment of epilepsy, nephrolithiasis was a recurrent issue affecting participants. In individual studies, the incidence rates of nephrolithiasis ranged from 0 to 25% [34]. A recently published meta-analysis of participants on a ketogenic diet showed a mean incidence of 5.9% in both children and adults after a mean follow-up period of 3.7 ± 2.9 years [30]. During the follow-up period, adults had a higher incidence of kidney stones of 7.9%. In contrast, the average annual incidence of nephrolithiasis in the general population is reported to be 0.25–0.3% per year [35]. It appears that adults treated with a ketogenic diet for epilepsy had an annual incidence of nephrolithiasis that is 7–8 times higher than the average population.

Although those on a ketogenic diet for epilepsy may have nondietary reasons for their nephrolithiasis, there is a growing body of theoretical evidence suggesting that the risk of nephrolithiasis may be largely attributable to the diet itself (Table 2). In addition, those using a ketogenic diet for nonepileptic reasons may already be at increased risk for nephrolithiasis due to reduced urinary pH from obesity and insulin resistance [36]. The acidosis that occurs with ketogenic diets has the potential to reduce urine citrate, which is a known inhibitor of stone formation [37]. Urinary citrate complexes with calcium in the renal tubule to reduce the concentration of urinary calcium, thus preventing the formation of calcium oxalate and calcium phosphate crystals [38]. In metabolic acidosis, citrate reabsorption is increased in the proximal tubule, leading to hypocitraturia.

**Table 2:** Dietary risk factors for nephrolithiasis with the ketogenic diet.

Risk Factor	Mechanism
Hypocitraturia	Metabolic acidosis from ketones
Hypocitraturia	Low fruit and vegetable consumption leading to low citrate levels
Hypocitraturia	Animal protein consumption leading to an increased dietary acid load
Hyperoxaluria	High dietary fat consumption, which binds to dietary calcium, leading to increased dietary oxalate absorption
Hypercalciuria	Metabolic acidosis, Low dietary phytate
Low urine volume	Reduced water intake from a low level of dietary fruits and vegetable

Another major risk factor for nephrolithiasis with a ketogenic diet is the high dietary fat content, which is integral for ketosis. The high amount of dietary fat may bind to dietary calcium in the intestine, preventing the absorption of dietary calcium and increasing the amount of oxalate absorbed [39–42]. The absorbed oxalate is ultimately excreted in the urine, increasing urinary oxalate and the risk of calcium oxalate nephrolithiasis. However, despite the potential for high amounts of calcium being bound to dietary fat, the ketogenic diet may also increase urinary calcium levels, which may be a result of bone buffering for metabolic acidosis.

An additional risk factor for nephrolithiasis is the reduced water consumption that occurs with the avoidance of fruits and vegetables, which are not only high in carbohydrates, but also water. Low levels of phytate consumption in a ketogenic diet may also contribute to increased urinary calcium binding to oxalate [43]. Phytate is commonly found in foods like grains and legumes that are restricted in the ketogenic diet. Finally, low urinary pH levels and high purine consumption can also promote uric acid stones.

## A PLANT-BASED KETOGENIC DIET FOR CKD

The ketogenic diet is an extreme version of a low carbohydrate diet, and low carbohydrate diets have been associated with an increase in mortality in a large meta-analysis [44]. In that same analysis, researchers found that the risk of mortality was decreased when carbohydrates were replaced with plant fats and plant protein, as opposed to animal fats and animal protein [44]. Based on this and other evidence supporting the use of plant foods, it is reasonable to consider the use of plant foods for those adopting a ketogenic diet. Plant foods have already been described to have several potential benefits for patients with kidney disease [18]. In addition, the alkali found in these foods may help prevent worsening of metabolic acidosis or the development of nephrolithiasis. The MUFAs and PUFAs often found in plant fats may also help prevent an increase in LDL-C. Examples of plant fats for consideration by patients with CKD on a ketogenic diet include avocados, oils (i.e. olive oil and canola oil), nuts and seeds.

## CONCLUSION

**Figure 1:**
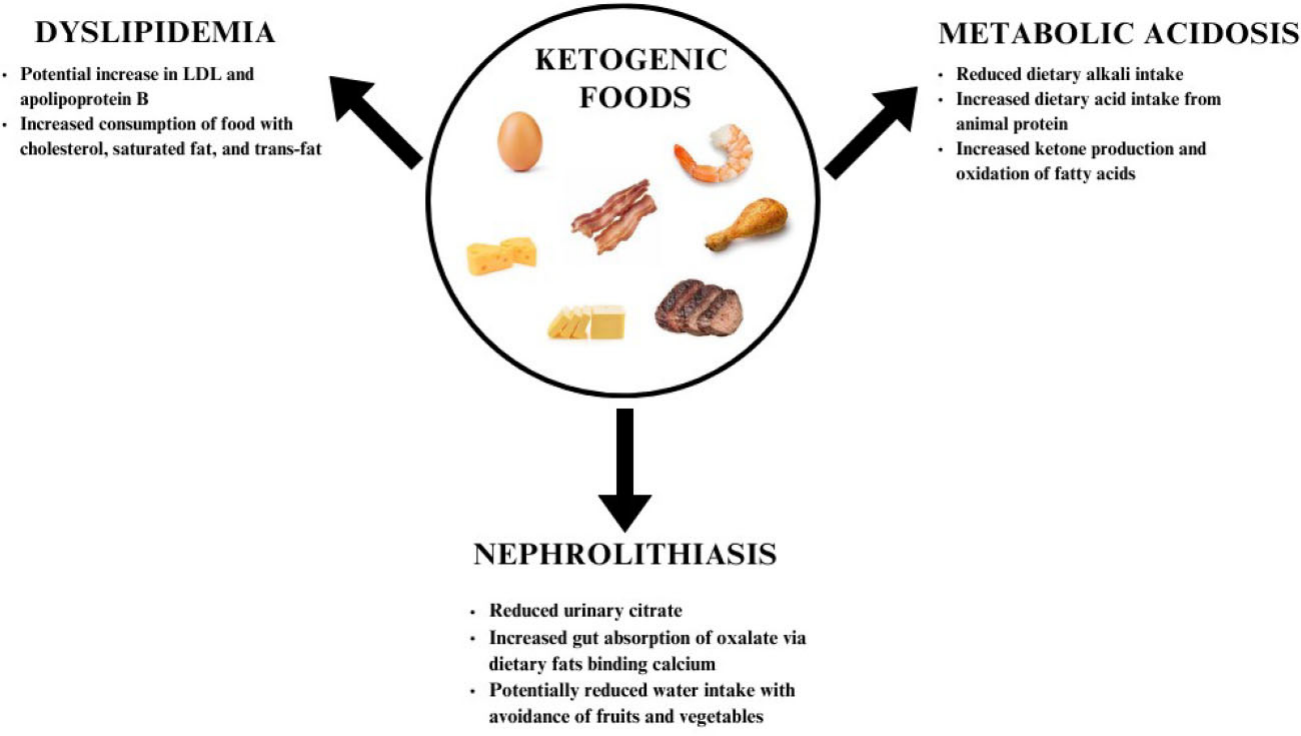
The potential risks of the ketogenic diet in CKD.

The ketogenic diet has the potential to cause drastic weight loss and substantial improvement in markers of glycemic control. However, adherence to the diet is difficult in dietary studies and, by 12 months, any benefit from the diet is rendered negligible over comparator, higher carbohydrate diets. Given the potential for side effects (including nephrolithiasis, metabolic acidosis and dyslipidemia; Fig. 1) and the abundance of alternative diets, it is reasonable for providers to consider the risks and benefits of the diet with patients. For those with CKD, the diet may exacerbate complications of kidney disease such as metabolic acidosis, which, if left untreated, has been associated with a more rapid decline in kidney function. Finally, many popular iterations of the ketogenic diet depend on animal-based foods and eschew carbohydrate-containing plant-based foods, which prevents patients with kidney disease from harnessing the potential benefits of plant-based diets, like in the PLADO diet. Given the potential for harm and numerous dietary alternatives, it is reasonable to reserve the ketogenic diet for those who have tried other dietary strategies. In addition, although unproven, a plant-based ketogenic diet may mitigate some of the concerns with animal-based versions of the ketogenic diet.

## Data Availability

No new data were generated or analyzed in support of this research.

## Notes

(See related article by Weimbs et al. Ketogenic metabolic therapy for chronic kidney disease - the pro part. *Clin Kidney J* (2024) 17: sfad273.)

(See related article by Zoccali. Moving on obesity treatment in CKD: inertia is unjustified. *Clin Kidney J* (2024) 17: sfad275.)
